# Cellular resistance mechanisms in cancer and the new approaches to overcome resistance mechanisms chemotherapy

**DOI:** 10.15537/smj.2023.44.4.20220600

**Published:** 2023-04

**Authors:** Hajir A. Al Saihati, Ali A. Rabaan

**Affiliations:** *From the Department of Clinical Laboratory Science (Al Saihati), Applied Medical College, University of Hafr Al Batin, Hafr Al Batin, and from the Depatment of Molecular Diagnostic Laboratory (Rabaan), Johns Hopkins Aramco Healthcare, Dhahran, Kingdom of Saudi Arabia.*

**Keywords:** cancer, chemotherapy, chemoresistant

## Abstract

Despite major advancements in cancer healing approaches over the last few decades, chemotherapy remains the most popular malignancy treatment. Chemotherapeutic drugs are classified into many kinds based on their mechanism of action. Multidrug resistance (MDR) is responsible for approximately 90% of fatalities in malignancy cases treated with standard chemotherapeutics or innovative targeted medicines. Many innovative prospective anti-cancer medicines displayed high anti-cancer efficacy in a single application. However, combining them with other medications improves cancer treatment efficacy. This supports the belief that a combination of drugs is significantly more effective than a single medicine. Due to the intricacy of MDR processes and the diversity of tumor illnesses, there will rarely be a single medicine that can be utilized to treat all types of cancer. Finding new medications that can reverse MDR in malignancy cells will augment efficacy of chemotherapeutic agents and allow us to treat cancers that are now incurable.

**M**alignancy is the second principal cause of death worldwide, accounting for roughly one out of every 6 deaths. Although numerous techniques for treating malignancy, including gene therapy, radiotherapy, immunotherapy, surgical resection, endocrinal therapy, and chemotherapy remains the most prevalent cancer treatment option. Traditional chemotherapy was the main member of anti-cancer treatment; nevertheless, its efficacy was vulnerable to acquired or intrinsic drug resistance. Understanding the mechanisms for different chemotherapeutic drug resistance has been critical to developing well therapeutic policies, perfectly causing a personalized drug schedule for enhanced therapeutic reactions and inhibiting the treatment with some previous ineffective chemotherapeutic agents. A range of chemotherapeutic drug resistance mechanisms have been well-documented, including general multi-drug resistance (MDR) agents and characteristics unique to one class of drugs. Most of these processes were first explored in tumor cell lines and then confirmed in the clinical setting. However, only a few criteria have been utilized in clinical practice as prognostic markers to determine the best treatment plan for a specific cancer patient undergoing standard chemotherapy.^1^

Targeted medications, on the other hand, have benefited from individualized therapy. The advancement of genomic, proteomic, transcriptomic, and screening technologies has led to a better understanding of the molecular pathways that cause specific malignant tumors to originate. Drugs have been developed based on these findings that target a pathway or protein stimulated in the malignant tumor. These have been stimulated kinases, like epidermal growth factor receptor (EGFR) in melanoma, B-Raf proto-oncogene, serine/threonine kinase (BRAF) in pulmonary cancer, and fms-like tyrosine kinase 3 (FLT3) in acute myeloid leukemia (AML) cases. Resistance mechanisms to several chemotherapeutic medicines have been studied widely in mice and cancer cell line models. Bypassing the blocked signaling route, enriched drug efflux via ABC superfamily multi-drug efflux transporters, down-regulation of the principal drug target, and chemical changes of medicines into non-effective metabolites are the main processes. Aside from integrating the ultimate understanding of drug resistance mechanisms into clinical practice, only a few of these mechanisms have been proven effective outside of the laboratory context (namely, in patients). In combination with the development of new drug resistance and molecular mechanisms, modern cancer genome sequencing may lead to the validation and identification of clinically relevant resistance mechanisms, allowing for an improved context for using personalized therapeutic regimens in the best treatment decisions for many malignancy cases.^1^

We will discuss the most up-to-date information on the cellular resistance mechanisms to chemotherapy, the chemotherapeutics utilized in treatment, and the mechanisms of action of new prospective anti-cancer medicines targeted to overcome these resistance mechanisms.

## Drug resistance in cancer chemotherapy

**Figure 1:**
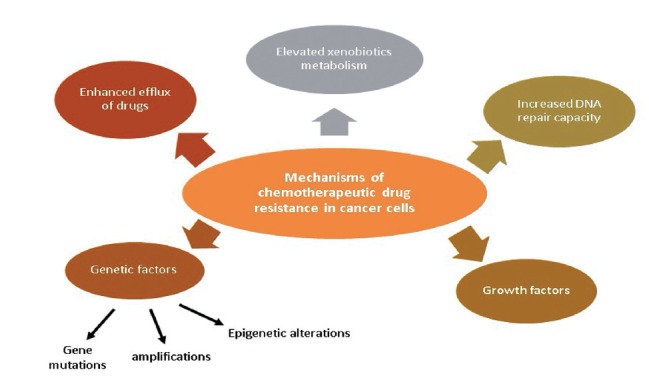
- Chemotherapeutic drug resistance mechanisms in cancer cells.^2^

Drug resistance is responsible for more than 90% of cancer-related deaths. Improved drug efflux, genetic elements (gene amplifications, mutations, and epigenetic alterations), greater deoxyribonucleic acid (DNA) repair capacity, growth factors, and increased xenobiotic metabolism are all possible reasons for MDR of malignant cells during chemotherapy (Figure 1). Each of these pathways results in a decline in the therapeutic efficacy of medications provided, posing additional challenges in cancer treatment.^2^

## Drug efflux enhancement

The modulation of excretion, absorption, and distribution of a diversity of chemical complexes has been attributed to ATP-binding cassette (ABC) proteins found in the cell membrane, such as P-glycoprotein (P-gp)/ATP-binding cassette subfamily B member 1 (ABCB1) or breast cancer resistance protein (BCRP). These proteins guard cells against death produced by high intracellular drug concentrations. However, they may also slow drug delivery by reducing intracellular concentrations, bioavailability, and migration across the blood-brain barrier (BBB). P-glycoprotein, considered an ATP-dependent drug efflux pump for xenobiotic compounds, has been extremely expressed on the surface of the endothelial cells contributing to the reduction in chemotherapeutic drug infiltration to particular places, particularly in the case of cerebral malignancies therapy where anti-cancer drugs have been commonly unable of passing through the BBB. In addition, tumor size had a significant impact on drug penetration. Chemotherapeutic drugs have proved less effective in huge tumors owing to inadequate blood flow, likened to tiny tumors with virtually limitless nutrition and oxygen delivery. The P-gp protein guards the brain against potentially harmful compounds while also restricting therapeutic substances responsible for increasing treatment complexity. The only way to overcome the barrier was to elevate the medication concentration, resulting in systemic toxicity. This is one of the reasons why increased drug efflux has been identified as one of the primary mechanisms of malignancy cell resistance to chemotherapy.^3,4^

Breast cancer resistance protein and P-gp could transport an extensive range of functionally and structurally disparate anti-cancer mediators to the extracellular space, including epipodophyllotoxins, anthracyclines, actino taxanes, bisantrene, imatinib, camptothecins, saquinavir, vinca alkaloids, thiopurines, colchicine, methotrexate, and mitoxantrone.^5^

Significant correlations between enlarged expression of P-gp in malignancy cells and greater resistance to etoposide, doxorubicin (DOX), olaparib, vinblastine, and paclitaxel have been discovered among various chemotherapeutics. P-glycoprotein overexpression has been seen in approximately half of the cases.^6^ The P-gp overexpression has been observed following anticancer agent introduction in some cancers, such as hematological malignancies like acute AML and acute lymphoblastic leukemia (ALL).^2^

Overexpression of BCRP and P-gp has been related to reduced clinical response and MDR in chronic lymphocytic leukemia, multiple myeloma, ALL, metastatic breast cancer, and AML.^5^ Furthermore, P-gp was found to have an important role in MDR in malignancy cells, facilitating intracellular chemotherapeutic drug efflux and suppressing tumor necrosis factor (TNF). Apoptosis-inducing ligands that are TRAIL-mediated in addition to caspase-associated are similar.^7^ Although P-gp inhibitors displayed improved efficiency in vivo and in vitro tests, the US Food and Drug Administration (FDA) has not accepted any of them for clinical usage in malignancy therapy.^8^ Instead, Nanayakkara et al^8^ proposed new P-gp inhibitors that could be useful in cancer treatment. Chemotherapeutics were co-administered with the composites against 2-dimensional MDR ovarian and prostate malignancy cells and 3-dimensional prostate malignancy micro-tumor spheroids. According to the researchers, cell motility, viability, and survival were significantly reduced. Furthermore, none of the tested P-gp inhibitors have been found hazardous or have P-gp transport substrates. Furthermore, validated components increased the number of reporter chemicals that are P-gp transport substrates and the cellular retention of anti-cancer medicines. Natural potassium ionophores like salinomycin are another example of innovative potential P-gp inhibitors. Guberovic et al^9^ discovered that only a few of the crown ethers tested were considerably more effective than the recognized P-gp inhibitor verapamil in sensitizing MDR cells to paclitaxel and adriamycin.

Furthermore, Liu et al^10^ discovered that joining DOX with ascorbic acid increased DOX sensitivity in human MDR breast cancer (MCF-7/MDR) cells in vivo and ex-vivo. As those investigators demonstrated, ascorbate increased cell reaction to DOX by stimulating cellular drug accumulation linked to the initiation of reactive oxygen species-dependent ATP reduction.

A new natural syncarpic acid-conjugated monoterpene, tometodione M (TTM), was also a molecule that could be employed in chemotherapy. The medication improved intracellular rhodamine 123 and DOX accumulation in human MDR leukemia cells (K562/MDR) and MCF-7/MDR cells by reducing P-gp-linked drug efflux. Tometodione M triggered MDR degeneration in cancer cells via inhibiting p38 mitogen-activated protein kinase (MAPK) signaling, which lowered P-gp protein and mRNA expression. Tometodione M also caused apoptosis and decreased colony formation in docetaxel-treated K562/MDR and MCF-7/MDR cells, increasing docetaxel cytotoxicity.^11^

Furthermore, Yuan et al^12^ discovered that cinobufagin, a material derived from the Asiatic toad’s posterior auricular glands and skin, influenced the intonation of P-gp activity in human P-gp-overexpressing colorectal carcinoma cells, involving Caco-2/ADR, HCT116/L, and LoVo/ADR, implying that it might be used in combination with chemotherapeutics. In MDR cells, cinobufagin greatly boosted intracellular accumulation of rhodamine 123 and DOX, as well as displaying apoptotic qualities. Furthermore, cinobufagin affected P-gp overexpression in LoVo/ADR cells by enhancing their sensitivity to P-gp substrate medicines such as DOX. Although additional research on the mechanisms of action of cinobufagin revealed no alterations in P-gp expression, cinobufagin was found to have a considerable influence on noncompetitive P-gp ATPase activity.

Furthermore, iso-pencillixanthone A (iso-PXA), which occurs naturally in the fungus Penicillium oxalicum, was identified as a chemical element that could be used in cancer chemotherapy. By activating P-gp ATPase and decreasing P-gp expression, iso-PXA might enhance the intracellular concentration of (VCR) in the human cervical cancer cell line HeLa/VCR. Iso-pencillixanthone A activated the intrinsic apoptotic pathway by activating caspase-3, caspase-9, and poly (ADP-ribose) polymerase (PARP). Moreover, the Iso-PXA prompted apoptosis by degrading the induced myeloid leukemia cell differentiation protein (Mcl-1) and accumulating the F-box and WD repeat domain-containing 7 pro-apoptotic proteins.

Chen et al^13^ investigated gallocatechin, catechin, taxifolin, luteolin, and human P-gp activity as a relationship between the actions of natural flavonoids. Taxifolin lowered ABCB1 expression and suppressed P-gp function via DOX efflux and noncompetitive rhodamine suppression in a concentration-dependent manner.

Quinidine was an eminent, FDA-approved medication utilized to treat arrhythmia, pseudobulbar affect, and malaria in clinical settings. However, the drug’s side effects connected to myocardial diseases, such as torsade de pointes and long QT syndrome (LQTS), make it difficult to use, for instance, a P-gp inhibitor in clinical practice. Snyder et al14 discovered that polymer-drug conjugates, for example, the methoxypolyethylene glycol (mPEG) glycine-quinidine conjugate, can help reverse MDR by decreasing P-gp. The tested compound not only suppressed P-job gp’s to the same extent as quinidine but also considerably reduced quinidine distribution in the mouse myocardial.^15^

Sitravatinib, a new receptor tyrosine kinase inhibitor, has been linked to the reversal of MDR in BCRP- and P-gp overexpressing malignancy cells. The examined chemical reduced the medication efflux activity of BCRP and P-gp in MDR cancer cells in a concentration-dependent way without affecting the protein expression of BCRP and P-gp. At submicromolar doses, sitravatinib reversed MDR facilitated by BCRP and P-gp.^5^

The cytotoxicity of cisplatin in P-gp overexpressing HepG2 cells was further investigated using new P-gp inhibitors, polyethylene glycol-modified titanium dioxide nanoparticles (PEG, TiO_2_, and NPs). Increased cisplatin cytotoxicity has been connected to the down-regulation of P-gp expression in HepG2 cells by TiO_2_, PEG, and NPs.^16^

## Influences of genetic factor

Tumor protein p53 (TP53) gene mutation, typically found in tumor cells, is one of the most recognized indicators of carcinogenesis. The remarkable importance of the TP53 gene in safe-guarding an organism against tumor progression and neoplastic transformation has been proven by Mantovani et al.^17^ The TP53 tumor suppressor is important for genome constancy and cellular homeostasis via orchestrating a variety of procedures and effector pathways, comprising cell cycle control, apoptosis induction, and G1 arrest in the event of genotoxic stress during duplication.

**Figure 2:**
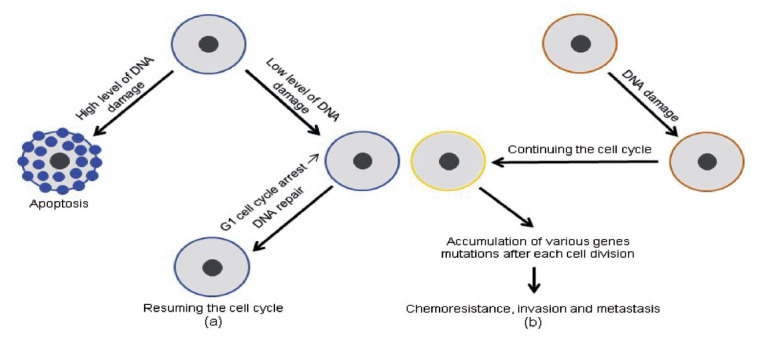
- Differences in TP53 gene expression between normal (a) and malignant (b) cells, as well as their consequences: (a) a precise level of TP53 gene expression and (b) a lowered level of TP53 gene expression.^17^

The TP53 pathway’s protective effect was reversed by activating infiltration, metastasis, and chemo-resistance when tumor-suppressive functions were lost due to missense mutations in the TP53 gene, which are particularly common in human malignancies. In most cases, anti-cancer medicines cause DNA damage, which leads to cell death due to TP53 stimulation. In contrast, malignancy cells that have lost their TP53 activity have been able to continue duplicating regardless of the type or extent of DNA damage, rendering them resistant to genotoxic medicines (Figure 2).

Inhibiting key enzymes in regulating cell propagation, such as dihydrofolate reductase, was a key role of several chemotherapeutics, including methotrexate. Because gene amplification is a possibility in 10% of malignancies, primarily leukemia, cancer cells may be able to overcome this repression by increasing gene transcription, which encodes the enzyme. This procedure has been linked to selective chromosome synthesis, which results in numerous copies of identical genes. Homogeneously stained areas or double-minute chromosomes have been used to identify these amplified sequences. Each of those genes has been transcribed to yield more mRNA, which is then utilized in the translation procedure to produce new enzymes. The medication concentration was restricted. It could not prevent the augmented amount of enzyme at times.^18^

**Figure 3:**
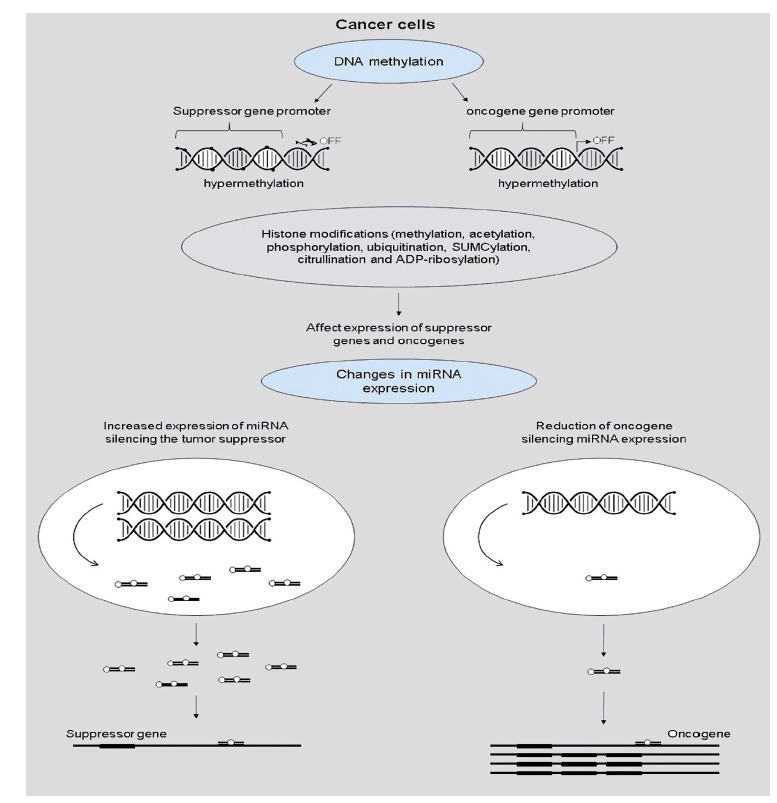
- Cancer cell gene regulation by epigenetic alterations.^19^

The most recent findings emphasize the critical significance of epigenetic changes in cancer cells as a cause of anti-cancer treatment resistance. Cancer development could be influenced by tumor suppressor genes silencing DNA hypermethylation or oncogene expression increased by DNA hypomethylation. The epigenome has undergone multiple alterations during carcinogenesis, including genome-wide DNA methylation loss, worldwide changes in histone modification marks, localized hypermethylation (especially in CpG promoter islands of tumor suppressor genes), and alterations in miRNA expression (Figure 3).^19^

## Influences growth factor

Ham et al^20^ revealed that there have been significant links between inflammation, malignancy development, and cancer development. The acute immune response aided tumor elimination, whereas the chronic immune reaction aided tumor development and infiltration. Compared to drug-sensitive cancer cells, MDR cancer cells have augmented autocrine formation of growth factors such as interleukin (IL)-1, IL-6, IL-4, and IL-8. Ham et al^20^ also confirm the link between IL-6’s impact on malignant-linked fibroblasts in the malignant stroma and the MDR of gastric cancer cells. The scientists discovered that IL-6 is a chromatin assembly factor-1 (CAF)-a specific secretory protein that confers chemoresistance to gastric cancer cells through paracrine signaling. Furthermore, the researchers discovered that using tocilizumab, a monoclonal antibody against the IL-6 receptor, inverted the CAF-mediated reduction of apoptosis in both in vivo and in vitro experimental settings. This research confirmed the potential therapeutic use of IL-6 inhibitors to improve anti-cancer drug sensitivity in gastric malignancy cells.

Malignancy chemoresistance could be raised not only by intracellular elements but also by elevated extracellular fibroblast growth factors found in the media of metastatic and solid cancers. Medications with varied mechanisms of action, involving DOX, paclitaxel, and 5-FU have been unsuccessful against malignancies with high levels of these extracellular factors to demonstrate the significance of fibroblast growth factors in cancer chemoresistance development.^21^

Glioblastoma is considered one of the most lethal brain cancers in adults and is characterized by obvious genetic heterogeneity. However, the modifications in receptor tyrosine kinase signaling activation have been amongst the most popular molecular modifications in glioblastoma. The critical link between signaling via the fibroblast growth factor (FGF) receptors and glioblastoma advancement has been recommended by Jimenez-Pascualel et al.^21^ Hence, inhibiting this signaling pathway using small-molecule inhibitors of FGF receptors, which are now being tested in glioblastoma treatment, could be a viable option.

Numerous information revealed that the amplified action of protein kinase C and extracellular matrix (ECM) in breast cancer cells have been linked to their chemotherapy resistance. It has been established that ECM showed a significant function in breast cancer invasion, development, and spread. Extracellular matrix remodeling was the main cause responsible for stimulating cancer spread and metastasis, particularly matrix metalloproteinases (MMPs), involving MMP-2, -9, -11, and -14, that destroy the matrix proteins. It has been suggested that D mannuronic acid, via inhibiting MMP-2 and -9, could be a potential anti-cancer agent. Nevertheless, other variables include ECM integrins b1-, b5-, and b6-; Hic-5 and ECM1 proteins; and enzymes such as LOXL2, LOXL4 heparanase, and procollagen lysyl hydroxylase-2 have been discovered to play a role in the control of breast cancer growth and progression. Additionally, stromal cells, comprising cancer-associated fibroblasts, adipocytes, and tumor-associated macrophages (TAMs), have been revealed to be related to tumor progression through a diversity of procedures (namely, generating a vessel network that helps in the nutrition of the cancer bulk as well as the secretion of vascular endothelial growth factor A (VEGF-A) via TAMs causing cancer infiltration).^22^

## Deoxyribonucleic acid-repair enhancement

Another factor that contributed to cancer cells developing resistance to a diversity of anti-cancer medicines was their ability to repair DNA damage. The nucleotide excision repair (NER) pathway’s DNA repair endonuclease XPF and DNA excision repair protein ERCC1 have been critical for the successful repair of DNA damage initiated by platinum-based compounds and cross-linking. It has been discovered that overexpression of the ERCC-1 and XPF and proteins is linked to the progress of cisplatin resistance in cancer cells.^23^

The failure of chemotherapy treatment was due to the truncated target specificity of a diversity of anti-cancer medicines previously established. Nonetheless, the successful use of PARP inhibitors against BRCA-deficient cancers has given rise to a new perspective on developing novel DNA repair protein inhibitors.^24^

ERCC1-XPF inhibitors have been identified in new combinations, for example, E-X PPI2 and E-X AS7. After using E-X AS7 or E-X PPI2, researchers discovered improved melanoma cell susceptibility to cisplatin, suppression of NER activity, and fewer ERCC1-XPF heterodimers in ovarian malignancy cells. Furthermore, one of the catechol-based ERCC1-XPF inhibitors (13 compounds) showed higher activity in NER and selectivity against deoxyribonuclease I and Flap structure-specific endonuclease 1 (FEN-1) in A375 melanoma cells, resulting in improved cisplatin activity.^24^

Through a multistep computational strategy, Gentile et al^23^ initiated putative alteration sites of F06, an inhibitor of the ERCC1-XPF. The improved IC50 value for suppressing ERCC1-XPF activity has been discovered in a case of B5 compound among the researchers’ analogs of F06. These findings demand further investigation and optimization; on the other hand, methodologies based on the investigators’ computational methodology could be utilized to develop novel ERCC1-XPF inhibitors.

Tolerance and repair of Pt-DNA injuries depend on the effectiveness of the homologous recombination (HR) pathway and NER. Documents showed that replication protein A (RPA) may be a promising novel target for chemotherapy. Replication protein A has been implicated in DNA recombination and replication, as well as the DNA-damage response (DDR), NER, and HR DNA repair pathways, such as a single-strand DNA (ssDNA)-binding protein.^26^

New RPA inhibitor compounds have been found to act against epithelial ovarian cancer (EOC) and NSCLC in vivo and in vitro models. In an in vivo model of NSCLC, one of the chemicals studied, TDRL-551 showed antitumor efficacy such as a solitary agent and in combination with Pt. Furthermore, the synergy between TDRL-551 and platinum has been discovered in both tissue culture models of EOC and xenograft.^27^

Furthermore, novel analogs of TDRL-551, a previously described RPA inhibitor, have been developed to increase physicochemical properties and anti-cancer potential. Compounds 43, 44, 45, and 46 have been recognized as chemical compounds with higher solubility, low micromolar RPA inhibitory action, and improved cellular absorption, indicating that they could be useful in developing novel chemotherapeutics.^26^

In NSCLC cells, the drug AZD6738 was an additional ATR kinase inhibitor that caused cell death or senescence. In NSCLC cell lines, AZD6738 boosted gemcitabine and cisplatin cytotoxicity while also increasing cisplatin anti-cancer properties in ATM-deficient NSCLC cells. In mice, ATR kinase inhibition induced by daily AZD6738 treatment for 14 days was well tolerated and augmented the therapeutic nature of cisplatin in xenograft models. In ATM-deficient lung malignancy xenografts, the combination of AZD6738 and cisplatin showed remarkable anti-cancer properties.^28^

DNA double-strand breaks (DSBs) in BRCA1-deficient breast cancer cells could only be repaired via the non-homologous end joining (NHEJ) pathway due to intermittent HR repair. Suppression of DNA-dependent protein kinases (DNA-PKcs) in the NHEJ and DDR pathways may be a potentially interesting target in BRCA1-deficient breast malignancy therapy for this reason.^29^ In BRCA1-deficient breast malignancy cell lines, Albarakati et al^30^ discovered a synergy between cisplatin and 2 highly specific DNA-PKcs inhibitors (NU7441 and NU7026).

After administration of AZD7648, a greatly selective DNA-PK inhibitor, effective DOX sensitizer, and irradiation (radiation-induced DNA damage), further deterioration in patient-derived xenograft animals was seen. Furthermore, combining AZD7648 with olaparib, an eminent PARP inhibitor, leads to apoptosis, cell development suppression, and improved genomic instability in ATM-deficient cells. Moreover, AZD7648 improved the efficiency of olaparib in both xenograft models and patient-derived xenografts, resulting in long-term tumor shrinkage.^29^

The technique of mutagenic translation synthesis (TLS) has been linked to the formation of MDR in malignancy cells in the case of chemotherapy that causes DNA damage. Wojtaszek et al^31^ discovered that the highly specific small-molecule inhibitor JH-RE-06 interferes with TLS action via disrupting mutagenic POL recruitment. In both cultured mice and human cell lines, combining JH-RE with cisplatin increased cisplatin-induced cytotoxicity. Yamanaka et al^32^ also discovered a link between the disruptive POL and better DNA-lesion chemotherapeutic effectiveness.

Translation synthesis DNA polymerase Rev1 mutations in malignancy cells increased TLS activity, which improved proliferating cells’ tolerance to DNA damage during duplication, resulting in increased survival. Inhibiting mutagenic Rev1/Pol-dependent TLS in cells, chemicals 4 and 5 sensitized human fibrosarcoma HT1080 cells to cisplatin. Supplementary tests established the compounds’ selectivity, establishing them as the first TLS inhibitors that target Rev1’s C-terminal domain (Rev1-CT).^33^

Deoxyribonucleic acid DSBs have been shown to create DNA damage response RNAs (DDRNAs), which are accountable not only for DDR management but also for DNA repair guidance, in a DROSHA and DICER-dependent manner. Enoxacin, a DICER activity booster, enhanced DDR signaling and DNA repair in cells exposed to ionizing radiations. Enoxacin stimulated the synthesis of DDRNAs at defective telomeres and chromosomal DSBs, which resulted in the buildup of TP53 at damaged sites and, Thus, the suppression of homologous recombination, resulting in more precise and closer non-homologous end-joining DNA repair. Unfortunately, enhanced DNA repair induced by enoxacin improved the survival of normal cells and malignancy cells treated with anti-cancer drugs, perhaps leading to these cells developing the MDR phenotype.^34^

## Influences of elevated metabolism of xenobiotics

Chemotherapy resistance is influenced by transporter molecules and enzymes involved in drug metabolism. Numerous studies have proposed that anti-cancer therapies might cause the stimulation and expression of cell-protective gene products. The detoxification of endogenous and foreign substrates has been aided by drug-metabolizing enzymes, which were an important aspect of phase I and II metabolism (xenobiotics).

Cytochrome (CYP) isoforms like CYP1A2, CYP1A6, CYP1B1, CYP2B6, CYP2C19, CYP2C9, CYP3A4/5, and CYP2D6 are necessary for phase I detoxification and drug metabolism. Overexpression of CYP1B1 has been detected in several malignancy cell types, and it has been shown to impact the biotransformation of chemotherapeutics like flutamide, mitoxantrone, paclitaxel, and docetaxel.^35^ Augmented expression of the CYP2A6 enzyme, elaborated in the anti-cancer drug metabolism such as fluorouracil, ifosfamide, cyclophosphamide, and aflatoxin, has also been seen in breast carcinoma tissues. Furthermore, cancer cells with substantially elevated expression of CYP2A7CYP1B1, and CP4Z1 were found to be more resistant to a variety of chemotherapeutics.^36^

Changed expression of enzymes elaborated in phase II of drug metabolism, comprising glutathione-S-transferases (GSTs), gamma-glutamyl transferases (GTs), thiopurine methyltransferases (TPMTs), uridine diphospho-glucuronosyltransferases (UGTs), and dihydropyrimidine dehydrogenases (DPDs) in cancer cells may improve their MDR. Kinase inhibitors like regorafenib, sorafenib, pazopanib, and lapatinib have been shown to suppress UGT activity, particularly UGT1A1. In contrast to the actions of lapatinib and pazopanib suppression of UGT1A1 by sorafenib and regorafenib has been linked to hyperbilirubinemia in patients. In addition, innovative UGT1A4 inhibitors that specifically improved cancer cell susceptibility to chemotherapeutic drugs revealed a new possible method for combating cancer MDR.^37^

The MDR has been overcome in malignancy cells with raised GT and GST expression using GT-activated arsenic-based prodrugs like darinaparsin and 4-(N-(S-glutathionylacetyl amino phenylarsonous acid) (GSAO) and GST-activated agents like nitrogen mustard. Natural flavonoid derivatives like baicalein, phloretin, baicalin, and phloridzin (at micromolar concentrations) have also been linked to GST inhibition. Other new GST enzyme inhibitors and chalcone derivatives, such as 4,40-diflurochalcone, 4-methoxychalcone, 20-hydroxy-4-methoxychalcone, 4-fluorochalcone and 40-hydroxychalcone, have been discovered.^38^

Wang et al^39^ have pointed out that The deletion of an F-box only protein 8 (FBX8), a major component of the SKP1-CUL1-F-box (SCF) E3 ubiquitin ligases, has been linked to colon tumorigenesis acceleration, according to Wang et al.^37^ Through the ubiquitination process, FBXB caused GSTP1 to be degraded, which slowed the growth of colorectal cancer.

The functions of glutathione (GSH) are linked to cellular redox equilibrium. The GSH both detoxifies xenobiotics and boosts MDR in malignancy cells. Compared to normal cells, Malignancy cells produce more reactive oxygen species (ROS). Cancer cells acquired an improved antioxidant defense system to control the heightened oxidant status due to their furious growth and enhanced metabolism. Multiple mechanisms of apoptosis in malignancy cells have been linked to changes in GSH levels.^40^

The GSH has been overexpressed in tumor tissues generated from liver, lung, breast, and colon, and malignancies when related to normal tissues. The GSH’s increased detoxification capacity in malignancy cells has been linked to reduced chemotherapeutic drug action.^41^

Cancer cells may become more sensitive to existing chemotherapeutics if the GSH antioxidant defense system has been compromised. It has been proposed that a slight reduction in GSH levels would be a useful technique for increasing cancer cell chemosensitivity. Reduced GSH precursor availability, increased GSSG levels, suppression of the GSH synthesis procedure direct conjugation with GSH, and campaign of cellular GSH outflow were all ways to decrease cellular GSH levels.^42^

## Molecular level of drug resistance in cancer

**Figure 4:**
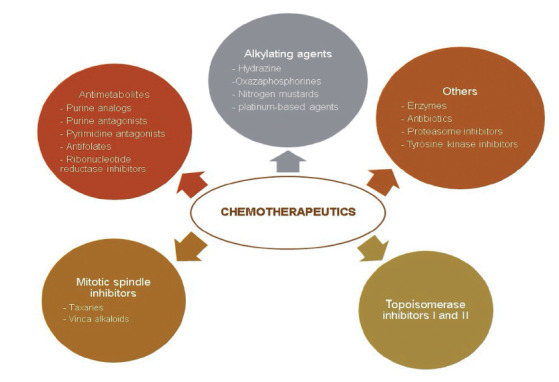
- Classification of commonly used chemotherapeutics depending on their mechanism of action.^43^

**Figure 5:**
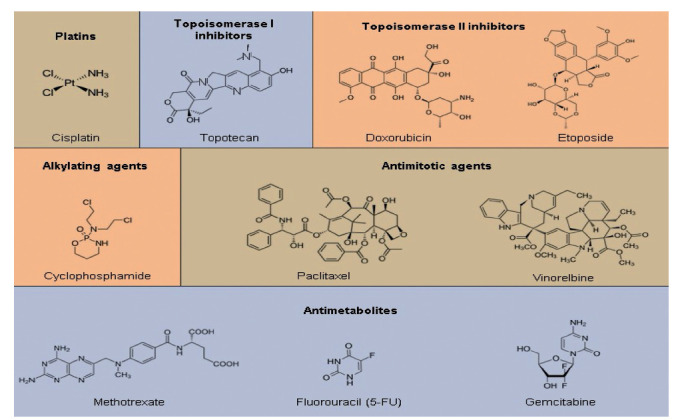
- Members of various chemotherapeutic classes have diverse chemical structures. There were 6 distinct chemotherapy classes, some of which are further subdivided into subcategories. The most often used drug’s structure has been displayed for each subclass.^43^

Chemotherapeutics could be classified into 2 groups based on their origin. Plant-derived (extracted from plants) or synthetic origins have also been possible. They have been classified as topoisomerase inhibitors, mitotic spindle inhibitors, alkylating agents, antimetabolites, and others based on their method of action (Figure 4 & 5).^43^

## Platinum-containing drugs

Platinum-based medications, like cis-diamine dichloroplatinum(II), were among the gold standard treatments for many cancers (CDDP or cisplatin). This vital class of anti-cancer drugs, which comprises the analogs oxaliplatin and carboplatin has been based on a platinum core that, when activated in the cytosol (through Cl substitution for OH), responds with numerous cellular structures, most remarkably guanine nucleobases, to generate intra- and inter-strand DNA cross-links. Even though platinum medications are successful in cancer treatment, their resistance has been common. Most resistance variables have been linked to DNA damage responses (DDR), implying that this was cisplatin’s primary method of action. However, other variables that target other processes in the cisplatin action cascade have been identified, donating to drug resistance to this medication.^44^

Platinum-based medications must come in tumor cells and accumulate in necessary amounts to target their macromolecular substrate (DNA). Multiple cisplatin-resistant cell lines had lower intracellular accumulation, and several uptake and export receptors were found. The subunits LRRC8a and LRRC8d of the volume-regulated anion channel (VRAC) help with cisplatin absorption and passive membrane diffusion.^45^ The damage to these VRAC subunits causes cisplatin and carboplatin resistance. Dissimilar to many other anti-cancer medications, cisplatin or its variations are not exported from cells through P-gp, but rather by MRP2/ABCC2, because MRP2 overexpression causes cisplatin resistance. Other drug resistance determinants which decrease the amount of active platinum-based drugs that form DNA adducts involve p22phox, which stops cisplatin from entering the nucleus, and enzymes that yield nucleophilic species like GSH, which converts hydroxylated cis-platin into an inactive glutathione-modified molecule.^46^

Even though these characteristics directly affect platinum medicines, there have been more general tumor escape mechanisms. Downregulation of the apoptotic factor FHIT, which has been frequently mutated in cancers, upregulation of the cell cycle regulator Dyrk1B and stimulation of many signaling pathways, counting p38MAPK and AKT, was among them. These features are likely to play a role in resistance to other kinds of chemotherapeutics also, and they can be more significant in resistance mechanisms to combination therapy than single platinum drugs.^47^

## Topoisomerase I inhibitors

Topoisomerase (Topo) I inhibitors (topotecan and irinotecan) and topoisomerase II inhibitors (teniposide, etoposide, and anthracyclines, such as DOX idarubicin, and daunorubicin) block topoisomerases’ activity in DNA replication, causing DNA strand breaks.^48^

Several medications have been industrialized to target Topo I, the most well-known of which are camptothecin and its analogs irinotecan and topotecan. Because Topo I is involved in comparable biological pathways during DNA transcription and replication, medications that target them cause similar patterns of DNA destruction in specific regions of the genome. Due to the partial redundancy of both topoisomerases, sequential therapy with Topo I and Topo inhibitors is being investigated to prevent resistance from the enhanced activity of the other topoisomerase.^49^

Camptothecin sensitivity has been controlled by modulators of Topo I activity, as shown by casein kinase 2. In experimental models, this kinase phosphorylates and activates Topo I, and its absence makes cells less sensitive to camptothecin. Drug resistance could also be caused by changes in the DDR pathway, resulting in enhanced DNA repair or a failure to initiate apoptosis. The DNA base repair pathway, which includes PARP1, TDP1, and XRCC1, has been the chief pathway for cells to repair Topo I inhibitor-dependent single-stranded DNA damage.^50^

Certainly, XRCC1 overexpression improved the camp-tothecin tolerance, while TDP1 deletion makes cells more sensitive. Likewise, PARP1 inhibition has been discovered to work in tandem with Topo I inhibition. Homologous recombination orchestrates the repair of DNA DSBs generated by camptothecins, as DSBs occur during replication in the S-phase, and cells lacking HR have been more susceptible to Topo I inhibitors. All pathways connected to resistance to Topo I inhibitors have been awaiting clinical justification, which has been compounded by the fact that Topo I inhibitors have been used as a portion of a multimodal regimen that also includes platinum medicines or Topo II inhibitors, both of which depend on DNA damage initiation.^51^

## Topoisomerase II inhibitors

Inhibitors of Topo II were a type of chemotherapeutic agent. Topoisomerase II unwound DNA and allowed one DNA strand to pass through another, followed by re-ligation of the broken strand by generating a DNA double-strand break. When the temporary DNA double-strand break occurs but before DNA ligation, Topo II poisons prevent Topo II in its active form, resulting in hazardous DNA double-strand breaks. Because multiplying cells, such as malignancy cells, regularly have high levels of topoisomerase II (the most common of the 2 isoforms) to adapt to more active DNA replication and transcription, Topo II inhibitors are thought to be more susceptible to inhibition in cancer cells than in normal cells.

Anthracycline-induced histone eviction inhibits DNA repair by removing histone H2AX, which is required for the DDR to begin, resulting in increased DNA damage and p53 activation. Histone eviction also changes the transcriptional landscape by removing epigenetic markers based on histone modifications. Such side effects can disrupt normal cellular physiology and add to the anti-cancer drug’s cytotoxicity. Daun favors an active chromatin area manifest by H3K4me3, and DOX favors an active chromatin area manifested by H3K36me3, whereas Acla (aclarubicin, an anthracycline that evicted histones but has not because DNA breaks) also targets repressive chromatin regions adorned with H3K27me3. Because intercalation of DOX into DNA has been adequate to promote histone eviction and has not necessitated active machinery, resistance strategies to anthracyclines and etoposide have largely focused on Topo II-mediated DNA break creation and repair.^52^

Anthracyclines’ cardiotoxic side effects, which accumulate in cardiomyocytes as doses rise, limit their usage in cancer patients. This means that many patients have not been treated further because of cardiotoxicity, although they would have responded to the medicine. Patients could, however, develop resistance to Topo II toxins. Drug efflux via the ABC transporter P-gp has been the most well-studied resistance mechanism for both etop and DOX. While P-gp played a crucial role in DOX resistance in rodents, its role in human cancer patients is less apparent. Moreover, P-gp transport inhibitors have demonstrated minimal therapeutic benefit in cancer patients treated with DOX, implying that other resistance mechanisms are more prevalent.^53^

Because anthracyclines are weak bases, they can be sequestered in lysosomes protonated in the acidic lumen. This substantially reduces nuclear drug exposure and efficacy. Treatment with bafilomycin A1, which inhibits vesicular ATPase, results in lysosomal alkalinization, which reduces drug buildup in lysosomes and restores drug sensitivity.^54^

Wijdeven et al^52^ revealed the SWI/SNF complex, a chromatin remodeler that loads Topo II onto DNA and is commonly mutated in malignancy, as a mechanism underlying DOX and etop resistance when inactivated. Tumors having this complex deleted or downregulated in the clinic showed a lower response to DOX-containing therapies showing that variables impacting Topo II activity can affect DOX sensitivity. By employing brief incubation times followed by wash-out trials with DOX to simulate clinical pharmacokinetics, this search also identified Keap1 and C9orf82/CAAP1 as factors implicated in resistance to Topo II toxins (as seen in patients). These function by slowing down DNA repair and controlling Topo II toxicity. Resistance to platinum-based medicines has also been linked to homologous recombination DNA repair regulators.^49^

Therefore, resistance mechanisms are similar to those identified in other drug classes and focus on DNA damage, subsequent repair, and cell viability management. Alternative anthracycline analogs may also aid in preventing drug resistance to Topo II inhibitors. Acla, for example, has been immune to at least some mechanisms that contribute to DOX resistance, such as alterations in the SWI/SNF complex.^49^

This was validated in AML patients, where patient’s refractory to DOX/daun-based chemotherapy had identical responses to an Acla-containing regimen as chemo-naive patients, implying that resistance to DOX/daun does not imply resistance to Acla. Furthermore, EZH2 mutations are found in a small percentage of diffuse large B-cell lymphomas, which increases H3K27me3 levels. Acla, which evicts the changed histones, makes these tumor cells more vulnerable.^49^

This was validated in AML patients, where patient’s refractory to DOX/daun-based chemotherapy had similar responses to an Acla-containing regimen as chemo-naive cases, implying that resistance to DOX/daun has not led to resistance to Acla. Moreover, EZH2 mutations are found in a small percentage of diffuse large B-cell lymphomas, which increases H3K27me3 levels. Acla, which removed those changed histones, has been more sensitive to these tumor cells.^49^

## Alkylating agents

Nitro mustards (chlorambucil, melphalan, and busulfan); hydrazine (temozolomide); platinum-based medications (oxaliplatin, cisplatin, and carboplatin); and novel, still-under-research off-on-type alkylating agents such vinyl-quinazolinone (VQ). Chemotherapeutics in this class form inter- or intra-strand crosslinks or transfer alkyl groups to DNA’s guanine residues, resulting in DNA mispair and preventing strand separation during DNA synthesis.^56^

On the other hand, alkylating agent side effects have been linked to CYP2B6. CYP2B6 expression can be used to determine the effective dose of alkylating drugs because higher p450 activity increases the rate of prodrug alteration and, thus, undesired toxicities without increasing treatment efficiency. After being hydroxylated, alkylating medicines enter cells via a flip-flop process and can be inactivated by aldehyde dehydrogenases, the most important being ALDH1. High levels of ALDH1 have been linked to a poor response to cyclophosphamide in breast cancer. ALDH1, on the other hand, was a marker for cancer stem cells and has been linked to chemotherapeutic response, implying that ALDH1 was not a specific sign for alkylating drug response. Glutathione can also modify alkylating drugs, but how this affects clinical responses has been unknown. Furthermore, P-gp (ABCB1) or MRP2 (ABCC2) might play a role in removing the drug from cells, but the importance of this in a clinical setting was unknown.^56^

## Antimetabolites

Antimetabolites could be classified into numerous groups: pyrimidine antagonists (gemcitabine, 5-fluorouracil (5-FU), capecitabine, and cytarabine), purine analogs (azathioprine, cladribine, and 6-mercaptopurine), purine antagonists (fludarabine), antifolates, ribonucleotide reductase inhibitors (hydroxyurea). Moreover, (pemetrexed, methotrexate, and pralatrexate), These anti-cancer medications disrupt critical metabolic pathways, disrupt DNA/RNA synthesis, or cause DNA strand breaks by inhibiting certain enzymes (ribonucleotide reductase, dihydrofolate reductase, and DNA polymerase) or incorporating incorrect structural analogs of pyrimidine/purine into DNA.^43^

Antimetabolite resistance was common; the most well-documented mechanism includes drug target mutation or increased expression. This entails overexpression of TS in the case of 5-FU and pemetrexed (PMX), which has been linked to a prognostic function in various cancers. The drug sensitivity of methotrexate is affected by overexpression or mutations in its target DHFR.^57^

Cancer cells, like other drugs, may reduce or enhance antimetabolite import and export. Methotrexate (MTX), PMX, and pralatrexate were all actively imported, but 5-FU was passively diffused across the membrane. Methotrexate and PMX are transported by the reduced folate carrier (RFC), but PMX was transported by both RFC and the proton-coupled folate transporter (PCFT), with the latter having a higher affinity. Reduced folate carrier expression or polymorphisms have been associated with MTX responsiveness in various tumor types. The relationship between response and mutation/expression of either RFC1 or PCFT has been less clear for PMX due to the redundancy of the 2 transporters.^59^

Numerous multidrug efflux transporters, such as ABCC11, ABCG2, and ABCC1-5, may aid drug export, albeit no clinical relationship between these transporters and clinical treatment responses has been established.^57^

Methotrexate, pralatrexate, and PMX were polyglutamylated by folylpolyglutamyl synthetase (FPGS) upon cellular entrance, increasing the retention of these antifolate polyglutamates because efflux transporters are no longer a substrate. Reduced FPGS expression, inactivating FPGS mutations, and overexpression of glutamyl hydrolase (GGH), which eliminates polyglutamate tails, lower cellular sensitivity to these medicines.^59^

In clinical studies, greater levels of polyglutamate-MTX were linked to improved treatment responses. Correlations between polymorphism and higher FPGS expression have also been discovered. Although GGH has a consequence on intracellular drug levels, a clinical link to treatment results has only been proven for one variant. This could be because GGH alters endogenous folates and stimulates their export, lowering DNA base biosynthesis.^60^

The TS inhibitors (PMX and5-FU) have been connected to dUTP metabolism, as TS blocking induces dTTP diminution and dUMP accumulation. By phosphorylating dUMP to dUTP, the nucleotide pool was moved from dTTP to dUTP. The BER pathway then repairs the dUTP misincorporation in DNA. The absence of uracil–DNA glycosylase (UDG), which removes dUTP from DNA, makes cells more sensitive to PMX and, to a lesser extent, 5-FU in vitro. High levels of dUTPase protect cells against 5-FU and PMX exposure by reducing dUTP concentration, which has been linked to poor colorectal cancer therapy outcomes.^57^

Reduced expression of genes tangled in the alteration of 5-FU to the active metabolite FdUMP, such as orotate phosphorylase transferase (OPRT), uridine monophosphate kinase (UMPK), and thymidine kinase (TK), as well as the overexpression of thymidine phosphorylase (TP) and dihydropyrimidine dehydrogenase (DPD), which converted the 5-FU into an inactive. Although the results were inconsistent, the levels of TP, TK, and DPD have been clinically related to the therapeutic effect, showing that these enzymes play only a minor role in 5-FU resistance.^57^

Resistance to gemcitabine works on the same principles as resistance to other antimetabolites. The nucleoside transporters hCNT1, hCNT3, and hENT1 were primarily responsible for cellular uptake of gemcitabine, with low hENT1 being associated with reduced overall survival of pancreatic cases after gemcitabine treatment. Other cancer kinds produced similar outcomes.^61^

Before its phosphorylation, gemcitabine might be changed into a metabolite released from cells by cytidine deaminase (CDA), and high levels of CDA have been connected to an unfavorable reaction in the clinic. On the other hand, people with low CDA levels were more likely to have side effects after receiving gemcitabine because the majority of CDA is formed in the liver.^62^

It is still unclear whether CDA could be utilized in tailored case selection or whether the dose of gemcitabine could be modified based on CDA levels. The enzymes NDPK, NMPK, and 5-Nucleotidase were also used to convert gemcitabine. However, no clinical link between drug resistance and these enzymes has been found. FdCTP, gemcitabine’s active form, competes with cytidine for DNA incorporation. Ribonucleotide reductase regulates the amount of cytidine in the cells (RR). Upregulation of its 2 subunits, RRM1 and RRM2, allows cytidine synthesis to outpace gemcitabine incorporation in vitro and in vivo, leading to gemcitabine resistance.^63^

**Figure 6:**
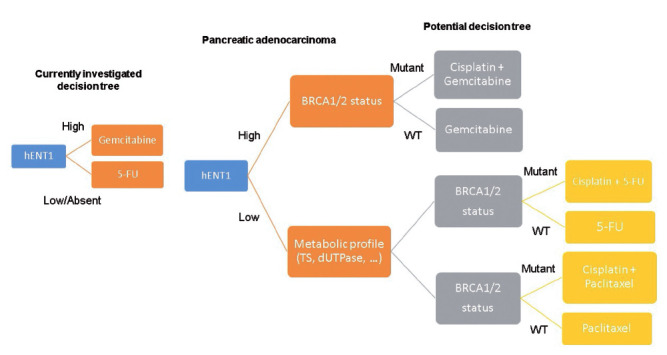
- A classification system could help with treatment decisions including classic anti-cancer drugs. Gemcitabine sensitivity in pancreatic malignancy is predicted by the expression level of the transporter hENT1. 5-FU is more effective in cancers with low hENT1 expression, while Gemcitabine is more effective in cancers with high hENT1 expression. BRCA1/2 mutations are found in pancreatic and breast cancers, and they render cancers more sensitive to cisplatin. Patients who are likely to respond to 5-FU can be identified by the expression of dUTPase, Thymidylate Synthase, or other factors associated in 5-FU resistance.^66^

Mlak et al^64^ found that cases with SNPs or changed RRM1 expression have lower gemcitabine effectiveness, while other studies have found no such link. Antimetabolites have the most well-confirmed presence of predicted resistance markers of all anti-cancer medication types. Various resistance factors work at 2 levels: intracellular accumulation and DNA base synthesis and incorporation. Currently, a clinical trial is evaluating the therapy of pancreatic patients based on hENT1 expression, which could lead to the first stratification criteria for conservative chemotherapy (Figure 6).

## Anti-microtubule agents

These were required for cell division’s cytoskeletal structure, signaling, transport, and chromosomal segregation. Several drugs interfere with microtubule dynamics because dividing cells are especially vulnerable to cytoskeletal disturbances. Spindle poisons bind tubulin and destabilize or stabilize microtubules (for example, taxanes and epothilones) (the Vinca alkaloids). Both drug classes function by blocking normal spindle development and triggering the spindle checkpoint. A lengthy pause causes death or increases mitotic slippage, in which cells exit the cell cycle without dividing. Due to their similar modes of action, both spindle poisons shared several resistance tactics, such as increased cell viability and drug removal via ABC drug transporters.^65^

Various mechanisms mediated resistance to tubulin-binding agents (TBAs), few of which were shared with other anti-cancer drug classes, such as spread through P-gp and upregulation of anti-apoptotic signaling pathways. In contrast, others, such as mutations in 1-tubulin and overexpression of microtubule-associated proteins, were specific to anti-microtubule agents (MAPs). Anti-microtubule drugs directly target 1-tubulin, and several mutations have reduced drug susceptibility.^65^

In clinical studies, the link between Tau expression and paclitaxel sensitivity has been ambiguous, with Bonneau et al^66^ reporting no significant effect of Tau.

Paclitaxel responsiveness and (phosphorylated) stathmin expression have an inverse connection in several tumor types. Cells could upregulate SYK kinase expression in response to paclitaxel therapy to break microtubules, most likely via phosphorylating tubulin and numerous MAPS. Paclitaxel’s efficacy in treating recurrent ovarian cancer may be harmed due to this.^67^

Survivin expression levels have been linked to disease progression and poor treatment response in various malignancies, counting breast malignancy and non-small cell lung cancer (NSCLC). In recent years, other mitotic factors, such as PDCD4, CASC1, and TRIM69, have been connected to paclitaxel sensitivity.^68^

Because cells die through apoptosis in response to TBAs, deregulation of survival signaling pathways critically affected chemosensitivity. This has been demonstrated to happen with the oxidative stress signaling pathway Keap1-Nrf2, HER2 signaling, Hippo signaling via TAZ, NF b signaling, and FAK1/YB-1 signaling.^47^

Inhibition of active signaling pathways could boost the effectiveness of anti-microtubule medicines, as proven in the combination of HER2inhibition (trastuzumab) and paclitaxel in experimental circumstances.^69^

The microtubule agent’s resistance was predicated on altered microtubule dynamics and their translation into a cell death program. Few characteristics have been definitively connected and proven in clinical trials, yet, given our current knowledge, MAPs can be the most probably predictive predictor of TBA resistance in patients.^70^

In conclusion, The development of MDR is a complex process associated with enhanced effux of drugs, elevated metabolism of xenobiotics, increased DNA repair capacity, growth and genetic factors, or any combination of these mechanisms. Knowledge of weak points of these mechanisms enabled scientists to develop new strategies against MDR cancer cells. Among novel potential anti-cancer agents, a remarkable part of these compounds demonstrated a strong anti-cancer activity in single application in both in vitro and in vivo studies. However, data has shown that their combination with other drugs significantly increased efficiency of cancer treatment. This confirms the current paradigm that combination therapy is considerably more efficient compared to any one drug on its own. Due to complicated nature of the mechanisms of MDR and heterogeneity of tumor diseases, probably, there will never be an individual drug which will find its use in every type of cancer treatment. This is the reason why further efforts to investigate the mechanisms of cancer drug resistance, especially identifying their currently unknown vulnerabilities, seems to be crucial in designing novel potential chemotherapeutics. Identifying new drugs that will be able to reverse MDR in cancer cells will increase the efficiency of commonly used chemotherapeutic agents, especially on the last stages of cancer development, and will give us an opportunity to treat currently incurable tumors.
